# The importance of gender equity in eye health leadership

**Published:** 2025-03-07

**Authors:** Jennifer Gersbeck, Kirti Prasad, Esmael Habtamu

**Affiliations:** 1 Executive Director – Influence and Scaling Impact: The Fred Hollows Foundation, Melbourne, Australia.; 2 Fiji Country Manager: The Fred Hollows Foundation NZ, Suva, Fiji Islands; 3 Chief Executive Director: Eyu-Ethiopia, Bahir Dar, Ethiopia; 4 Assistant Professor: International Centre for Eye Health, LSHTM, London, UK.

**Including women in eye health leadership improves health outcomes for everyone.**

Despite making up over 70% of the global health workforce, women hold only 25% of leadership positions in health care worldwide.^1^ This significant underrepresentation means that health initiatives are not yet fully benefiting from the diverse knowledge, perspectives, and expertise that women bring to leadership.^2^

Achieving gender equity in health leadership – i.e., including women at the decision-making level – improves health outcomes for everyone:

- As leaders, women understand the lived experience and challenges faced by women and girls in the community, who are often more underserved than their male counterparts.
- The insight and lived experience women bring to leadership can have a major impact on the productivity and retention of a predominantly female health workforce.
- As leaders, women often expand the health agenda, strengthening social inclusion and health for all.^1^

## The impact of women in leadership: evidence from the COVID-19 pandemic

One compelling example of the benefits of female leadership can be seen in the global response to the COVID-19 pandemic. Countries led by women achieved lower COVID-19 mortality rates compared to those led by men.^3^ For example, by June 2020, women-led countries like Germany and Aotearoa (New Zealand) reported fewer than 10 COVID-19 deaths per 100,000 people, whereas mortality in many other countries continued to increase at an alarming rate.^4^

**Figure:**
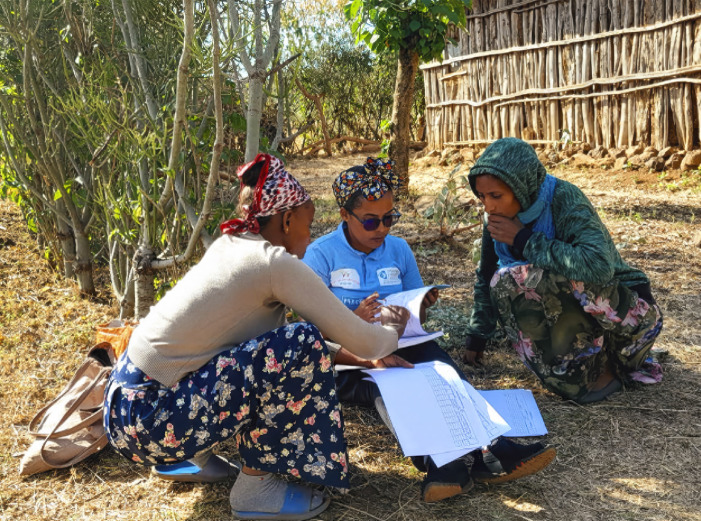
Marnat Adugna (centre), speaking with health extension workers (HEWs). ethiopia

© XXXXXXXXXXXXXXXX CC BY-NC-SA 4.0

Common characteristics of women leaders identified during the COVID-19 response were:

- **Their effective leadership and rapid response**, which helped to contain the virus.
- **Their emphasis on social inclusion.** Prioritising policies that addressed the social and economic impact of the pandemic helped to reduce the effect that COVID-19 had on vulnerable members of society.
- **Clear communication** from women leaders, as well as empathy where appropriate, allowed the public to understand the pandemic as well as the new policies.

These characteristics of women leaders are synonymous with the findings of an analysis that compared the key leadership capabilities of women and men, and found that women leaders excelled in taking initiative, acting with resilience, displaying integrity and honesty, inspiring and motivating others, and championing change.^5^

## Women bringing care to the community

Women eye health leaders play a major role in providing equitable access to eye care for their communities. An example of this is provided by Marnat Adugna, a health programme leader at Partners in Education Ethiopia, a charity organisation primarily engaged in improving access and quality of education and promoting health and well-being to disadvantaged communities in Ethiopia. Starting in 2019, Marnat led the development and implementation of successful community- and school-based eye health projects, including the establishment of six vision centres in primary health care facilities across Amhara region, outreach screenings and surgical campaigns in remote rural communities, and the training of hundreds of community health workers (CHWs) to integrate primary eye health services into the country's primary health care system.

Marnat explains: “I was inspired by the urgent need to address gaps in equitable access to eye health services in disadvantaged communities. As a health programme lead, I saw the opportunity to integrate eye health into existing community health structures, like the community health workers programme. I wanted to ensure that even the most remote communities could access essential eye care, which I believe is critical for improving overall health and educational outcomes.”

By the end of 2024, this work had resulted in eye screening for more than 90,000 students, teachers and community members, eye services for 32,000 people, and the distribution of over 3,500 pairs of spectacles.

**Women's leadership in the Pacific region**

Despite a long history of women's leadership in the Pacific region, women are still underrepresented in political and organisational leadership positions compared to global standards.^7^

For women in this region, it can be difficult to lead due to cultural norms and expectations that limit women's public roles and their participation in decision making.

However, when women in the Pacific do find opportunities to lead, they approach leadership with a collective mindset. “Women in the Pacific never sit at a table without representing their community,” says Dr Audrey Azuma, whose appointment as chief executive officer (CEO) of Fred Hollows New Zealand challenged traditional gender roles in the region. “They do not go into leadership roles as a journey of leadership and ambition on their own, but think more broadly about the difference they make for those sitting behind them.”

**Figure:**
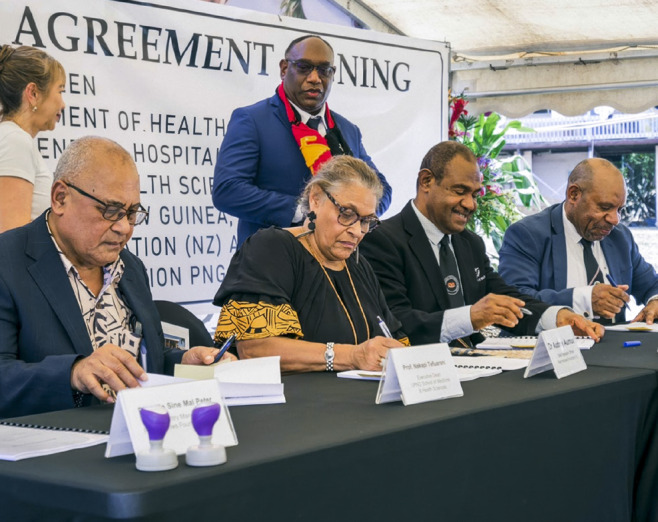
Dr Audrey Aumua (centre), CEO of The Fred Hollows Foundation New Zealand, signing an agreement with the National Department of Health. papua new guinea

© FHF NEW ZEALAND CC BY-NC-SA 4.0
